# Significance of Neutrophil Gelatinase-Associated Lipocalin Level-to-Serum Creatinine Ratio for Assessing Severity of Inflammation in Patients with Renal Dysfunction

**DOI:** 10.1155/2015/791926

**Published:** 2015-09-28

**Authors:** Jong Weon Choi, Tatsuyoshi Fujii, Noriyoshi Fujii

**Affiliations:** ^1^Department of Laboratory Medicine, College of Medicine, Inha University, Incheon 22332, Republic of Korea; ^2^School of Medicine, University of Tsukuba, Ibaraki, Japan; ^3^Department of Electrical Engineering and Bioscience, Waseda University, Tokyo, Japan

## Abstract

The aim of this study was to assess the significance of the neutrophil gelatinase-associated lipocalin/serum creatinine ratio (NGAL/sCr ratio) in patients with renal dysfunction. The percent difference between plasma NGAL level and the NGAL/sCr ratio was 36.7% (95% CI, 18.4–83.7%) in patients with sCr level ≥ 1.2 mg/dL. In a multivariate analysis, high sensitivity C-reactive protein (hsCRP) was significantly associated with the NGAL/sCr ratio and plasma NGAL level (*r* = 0.526 and *r* = 0.453, resp., *P* < 0.001). In a receiver operating characteristics curve, the diagnostic ability of the NGAL/sCr ratio to identify hsCRP > 4.0 mg/dL was superior to that of NGAL [0.783 (95% CI, 0.674–0.892) versus 0.733 (95% CI, 0.615–0.852), *P* = 0.032]. The area under the curve of the NGAL/sCr ratio was larger than that of hsCRP to detect corrected erythrocyte sedimentation rate > 25 mm/h and the neutrophil-to-lymphocyte ratio >4.5 in renal dysfunction. In short, the NGAL/sCr ratio may offer useful information when screening patients with both systemic inflammation and renal dysfunction.

## 1. Introduction

Neutrophil gelatinase-associated lipocalin (NGAL), also known as lipocalin 2, siderocalin, uterocalin, or oncogene 24p3, is a 25 kDa glycoprotein which was originally identified in cultured mouse kidney cells infected with simian virus 40 [1]. NGAL is one of the most promising new markers of renal epithelial injury. In contrast to serum creatinine, NGAL expression is induced specifically in the damaged nephron [2]. However, NGAL has important limitations, including its responsiveness to a variety of inflammatory conditions [3].

Determining inflammatory status, typically from bacterial or viral infection, is one of the most common problems in clinical practice. Inflammation is a known risk factor for decreased renal function [4]. Systemic inflammation contributes to the development of acute kidney injury, which is associated with the pathogenesis, such as infiltration of immune cells, apoptosis, microvascular thrombosis, and hypoperfusion [5, 6].

Although NGAL is a parameter for acute kidney injury, a wide heterogeneity in its predictive value is reported [7, 8]. Some research shows that plasma NGAL can increase in the absence of tubular damage and therefore should be used with caution as a marker of acute kidney injury [9]. It is unclear whether NGAL plays a more crucial role as an indicator of acute kidney injury than as an inflammatory marker. Furthermore, it is difficult to interpret the implications of increased NGAL levels, particularly in patients with both systemic inflammation and renal impairment.

Few studies have closely examined an adjusted value for plasma NGAL concentrations in regard to kidney function in inflammatory patients with renal dysfunction. In the current study, we determined a new parameter, the NGAL-to-serum creatinine ratio (NGAL/sCr ratio), and tested the use of the NGAL/sCr ratio in patients with inflammatory diseases and concurrent renal impairment, especially compared with NGAL and high sensitivity C-reactive protein (hsCRP).

## 2. Materials and Methods

### 2.1. Study Populations

A total of 162 patients under clinical investigation of systemic inflammation were assessed by measuring NGAL, sCr, the NGAL/sCr ratio, estimated glomerular filtration rates (eGFR), hsCRP, and the neutrophil-to-lymphocyte ratio (NLR). Patients ranged in age from 32 to 81 years (median age, 63 years), and 86 patients were male (53.1%). Age- and sex-matched healthy subjects (*n* = 45) with no evidence of inflammation or renal impairment were enrolled as a control group.

Medical records were reviewed for clinical and demographic data. Patients with multiple trauma (*n* = 3), stroke (*n* = 2), or cardiovascular diseases (*n* = 2) were excluded in this study because these conditions may influence plasma NGAL levels. Subjects who had missing values (*n* = 4), a recent operation (*n* = 2), or administration of drugs (*n* = 1) were also excluded. This study was approved by the Institutional Review Board of Inha University Hospital.

### 2.2. Measurement of Parameters

Plasma NGAL levels were measured by fluorescence immunoassay using the Triage NGAL assay (Alere, Inc., San Diego, CA, USA), which can rapidly analyze plasma NGAL with a measurable range from 15 ng/mL to 1300 ng/mL. The intra-assay CVs (*n* = 20) for three samples (mean NGAL, 75–516 ng/mL) were 4.3–6.2%; the interassay CVs calculated from duplicate results in 10 subsequent assays were 4.5–6.7%. An increase in the NGAL concentration above the medical decision point (150 ng/mL) was regarded as positive [8].

The NGAL/sCr ratio was calculated using the following formula: NGAL/sCr ratio = plasma NGAL level (ng/mL)/sCr concentration (mg/dL). For patients with sCr < 1.0 mg/dL, 1.0 mg/dL of sCr was used to avoid a falsely elevated ratio due to a decimal fraction. The NLR value was computed by the following equation: NLR = neutrophilic leukocyte counts (×10^9^/L)/lymphocyte counts (×10^9^/L).

ESR was determined by the Westergren sedimentation technique using StaRRsed Auto-Compact (Mechatronics Manufacturing BV, Zwaag, Netherlands). The corrected erythrocyte sedimentation rates (cESR) were calculated based on a normal hematocrit of 45% from the following formula: cESR (mm/h) = (subject's hematocrit/45) × ESR (mm/h). Blood urea nitrogen, sCr, and hsCRP levels were analyzed with a chemical analyzer (Hitachi 7600; Hitachi, Tokyo, Japan).

An increase in the levels of hsCRP, cESR, and NLR was defined as >4.0 mg/dL, >25 mm/h, and >4.5, respectively, which were the provisional cutoff limits based on the median values of the corresponding parameters in patient populations included in this study. Subjects were categorized into 2 groups: patients with renal impairment (*n* = 69) and without renal impairment (*n* = 93). The eGFR was calculated using the Modification of Diet in Renal Disease (MDRD) formula: eGFR = 186 × [sCr (mg/dL)]^−1.154^ × [age (years)]^−0.203^ [10].

### 2.3. Statistical Analysis

Data were expressed as mean ± standard deviation (SD) if they were normally distributed and as median (interquartile range) if nonnormally distributed. Categorical variables were listed as frequencies and proportions. A Mann-Whitney *U* test and Student's *t*-test were used to analyze data between the two groups. A multivariate regression analysis of NGAL and the NGAL/sCr ratio was conducted after adjusting for potential confounders. A receiver operating characteristics (ROC) curve was analyzed to compare the diagnostic ability of NGAL level and NGAL/sCr ratio to identify an increased hsCRP > 4.0 mg/dL. Additionally, the diagnostic value of the NGAL/sCr ratio and hsCRP for detecting NLR > 4.5 and cESR > 25 mm/h was investigated. A data analysis was done using SPSS software (version 14.0, SPSS Inc., Chicago, IL, USA). All *P* values < 0.05 were considered statistically significant.

## 3. Results

### 3.1. Baseline Characteristics of the Study Population

Of the 162 patients with inflammation, 69 (42.6%) had renal dysfunction with sCr ≥ 1.2 mg/dL. An elevated NGAL level > 150 ng/mL was observed in 56.8% of patient populations, which significantly exceeded the value of controls (0.0%, *P* < 0.001). Plasma NGAL levels and NGAL/sCr ratios were significantly higher in patients with inflammation than in healthy individuals (186.5 ng/mL and 147.2 versus 60.0 ng/mL and 58.0, resp., *P* < 0.001). However, there were no significant differences in cardiac biomarkers and systolic blood pressure between the two groups (Table 1).

### 3.2. Inflammatory Parameters and Kidney Function

Among subjects with inflammation, NGAL and the NGAL/sCr ratio were significantly elevated in patients with renal dysfunction versus without renal dysfunction. The percent difference between plasma NGAL concentration and the NGAL/sCr ratio was 36.7% (95% CI, 18.4–83.7%) in patients with renal dysfunction; however, no significant difference was observed in those without renal dysfunction. Levels of hsCRP, sCr, and NLR were increased to a significantly greater extent in the renal dysfunction group than in the comparison group; however, no significant difference was noted in neutrophilic leukocyte counts between the two groups (Table 2).

### 3.3. Regression Analysis

**Figure 1:**
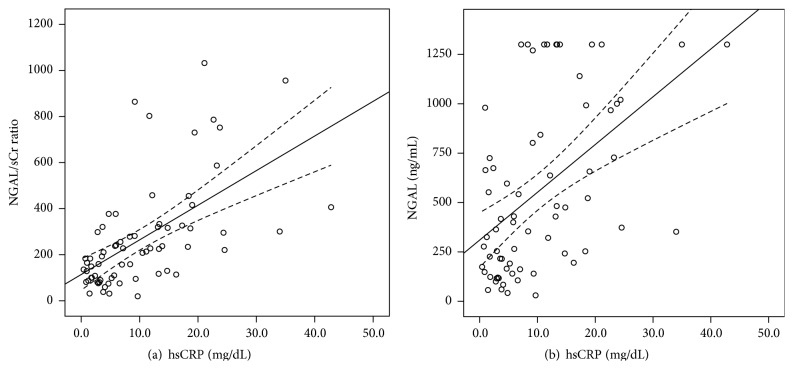
Scatter plots showing the correlation between hsCRP levels and the values of the NGAL/sCr ratio (a) and NGAL (b) in patients with renal dysfunction. The NGAL/sCr ratio significantly correlates with hsCRP (*y* = 15.027*x* + 114.26, *r* ^2^ = 0.349; *P* < 0.001) and plasma NGAL concentration (*y* = 24.175*x* + 310.19, *r* ^2^ = 0.237; *P* < 0.001). NGAL, neutrophil gelatinase-associated lipocalin; NGAL/sCr ratio, NGAL-to-serum creatinine ratio; hsCRP, high sensitivity C-reactive protein.

In a multivariate regression analysis adjusted for confounders, such as age, BMI, systolic blood pressure, hemoglobin, and troponin-I, the correlation coefficients of the NGAL/sCr ratio versus inflammatory parameters were slightly higher than those of NGAL versus inflammatory parameters, although no statistically significant differences were observed between the two groups (Table 3). A linear regression of NGAL and the NGAL/sCr ratio in relation to hsCRP level is presented in Figure 1. After adjusting to the sCr level, the scatter plot of the NGAL/sCr ratio converged more toward the correlation line than did the scatter plot of NGAL.

### 3.4. ROC Curve Analysis

**Figure 2:**
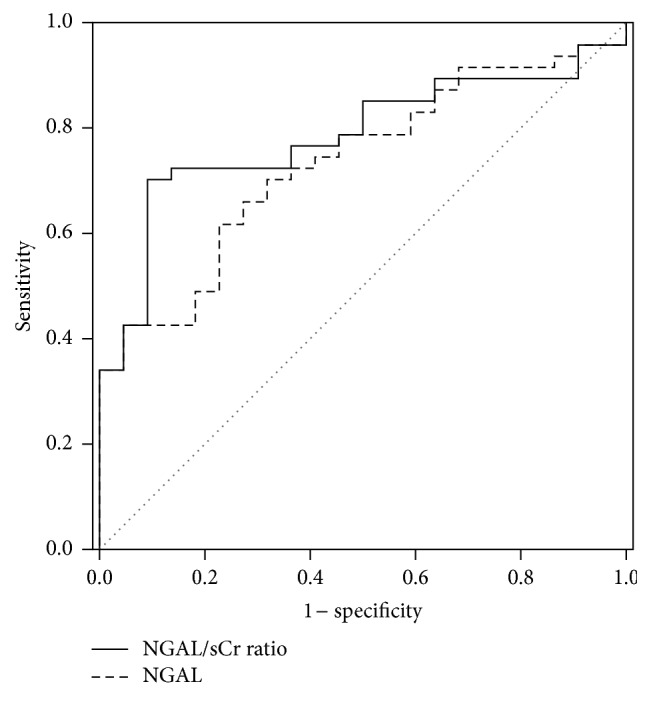
Comparison of the diagnostic values between NGAL and the NGAL/sCr ratio to identify hsCRP > 4.0 mg/dL in inflammatory patients with renal dysfunction. The AUC of NGAL/sCr ratio is significantly larger than that of NGAL [0.783 (95% CI, 0.674–0.892) versus 0.733 (95% CI, 0.615–0.852), *P* = 0.032]. NGAL, neutrophil gelatinase-associated lipocalin; NGAL/sCr ratio, NGAL-to-serum creatinine ratio.

**Figure 3:**
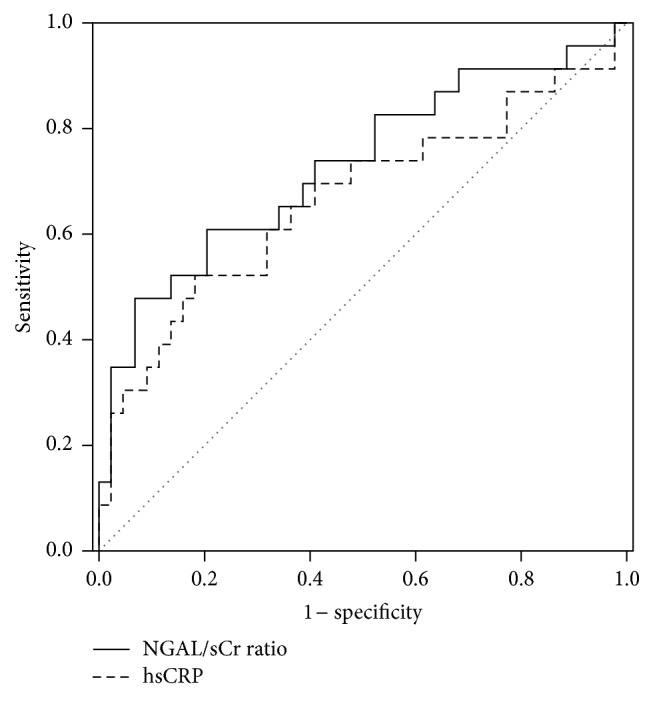
An ROC curve analysis shows the diagnostic ability of the NGAL/sCr ratio and hsCRP to detect an increase of NLR > 4.5 in patients with renal dysfunction. The AUC of the NGAL/sCr ratio is significantly larger than that of hsCRP [0.729 (95% CI, 0.594–0.865) versus 0.665 (95% CI, 0.517–0.815), *P* = 0.029]. NGAL/sCr ratio, NGAL-to-serum creatinine ratio; hsCRP, high sensitivity C-reactive protein.

The diagnostic values of NGAL and the NGAL/sCr ratio to identify hsCRP > 4.0 mg/dL and NLR > 4.5 in patients with renal dysfunction were investigated. In an ROC curve analysis, the AUCs of the NGAL/sCr ratio were significantly larger than those of NGAL [0.783 (95% CI, 0.674–0.892) versus 0.733 (95% CI, 0.615–0.852), *P* = 0.032; and 0.729 (95% CI, 0.594–0.865) versus 0.665 (95% CI, 0.517–0.815), *P* = 0.029, resp.] (Figures 2 and 3). The cutoff limit of the NGAL/sCr ratio to predict an increase of hsCRP > 4.0 mg/dL was 209.3, where the sensitivity and specificity of the NGAL/sCr ratio were 69.5% and 81.4%, respectively. Additionally, the diagnostic abilities of NGAL and the NGAL/sCr ratio for the various inflammatory parameters are listed in Table 4. The AUC of NGAL/sCr ratio was larger than that of hsCRP to detect NLR > 4.5 and cESR > 25 mm/h in patients with renal dysfunction.

## 4. Discussion

In this study, the NGAL/sCr ratio, a corrected value for plasma NGAL level, was investigated in patients with inflammation. The ratio was used to screen for the inflammatory status of patients with renal impairment and compared with the well-established parameters, such as NGAL and hsCRP. Our result shows that the new parameter accurately reflects the severity of inflammation in patients with impaired kidney function, particularly under inflammatory conditions.

Lindberg et al. [11] reported that neutrophilic leukocyte count was the main determinant of plasma NGAL level in a randomly selected general population. In our study, plasma NGAL, NGAL/sCr ratios, and hsCRP levels in inflammatory patients with renal dysfunction were significantly higher than in subjects without renal dysfunction; however, there was no significant difference in neutrophil counts between the two groups. Plasma NGAL concentrations were significantly correlated with hsCRP levels, but not with neutrophil counts in multivariate analysis. These results imply that neutrophilic leukocytes do not necessarily contribute to elevated NGAL concentrations, at least in inflammatory patients with renal dysfunction. These discrepancies may reflect differences in the severity of disease, inflammatory status, and kidney function in the subject populations among studies.

Recently, a novel inflammatory marker, NLR, has been proposed as an indicator of systemic inflammation [12]. Several studies have suggested that an altered NLR has prognostic value in chronic kidney disease, cardiovascular disease, and various malignancies [13]. Turkmen et al. [14] reported that NLR can predict inflammation in patients with renal disease. In our study, NLR was significantly increased in patients with renal dysfunction versus those without renal dysfunction. Additionally, NLR had a significant association with NGAL and the NGAL/sCr ratio; however, neutrophil count had no significant association with the corresponding parameters after adjusting for confounders. It is assumed that NLR correctly represents the inflammatory status in renal dysfunction. Our data support the results of Okyay et al. [15], which suggest that NLR provides significant information regarding inflammation in chronic kidney disease.

A group of investigators reported a strong positive association between NGAL concentration and hsCRP independent of age, sex, and adiposity, indicating that NGAL is an inflammatory marker [16]. Xu et al. [17] demonstrated that NGAL was more specific and sensitive than hsCRP in the discrimination between bacterial and viral infection. Smertka et al. [18] assert that increased plasma NGAL values are not solely a marker of acute kidney injury and more significantly represent inflammatory status. In our study, the diagnostic ability of the NGAL/sCr ratio to assess the degree of inflammation was investigated.

The AUC of the NGAL/sCr ratio was significantly larger than that of hsCRP to detect NLR > 4.5 in inflammatory patients with renal impairment. Additionally, for identifying hsCRP > 4.0 mg/dL, the NGAL/sCr ratio displayed a significantly larger AUC than NGAL. Similar findings were also observed with regard to cESR > 25 mm/h. These results suggest that the diagnostic value of the NGAL/sCr ratio is superior to that of hsCRP in patients with renal impairment, and the NGAL/sCr ratio may be a more reliable indicator than NGAL for identifying severity of inflammation.

In our multivariate model, the regression analysis revealed that hsCRP level has a slightly higher correlation coefficient with the NGAL/sCr ratio compared to NGAL, although no statistically significant difference was noted. A scatter plot of NGAL/sCr ratios centralized more into the regression line of hsCRP than did those of NGAL. A possible explanation for these findings is that elevated plasma NGAL concentrations, which are due to concomitant renal impairment, may be corrected by adjusting with sCr levels.

In fact, in our study, the median percent difference between NGAL and NGAL/sCr ratio was 36.7% in inflammatory patients with renal dysfunction, suggesting that impaired renal function may be responsible for approximately 36.7% of the increase in plasma NGAL concentration in patients with renal dysfunction under inflammatory conditions. Our results emphasize that plasma NGAL levels need to be amended with sCr levels to assess inflammatory status, particularly when impaired kidney function is in conjunction with inflammatory diseases.

Systemic inflammation is commonly accompanied by decreased kidney function [19, 20]. The NGAL/sCr ratio may reduce the influence of impaired kidney function on the plasma NGAL concentration when screening for inflammatory status in patients with decreased renal function. Based on our results, which were obtained in a cohort composed mainly of patients with inflammation and renal dysfunction, the NGAL/sCr ratio shows greater promise in diagnostic performance than NGAL.

This study has several limitations. We did not conduct serial measurements of plasma NGAL concentration. Because this study was a cross-sectional analysis, we could not draw a cause-and-effect relationship from our findings. As in any observational study, there may be unmeasured confounders for which we did not adjust during statistical analysis. Despite these limitations, our study demonstrates significance. To our knowledge, this is the first study to investigate the NGAL/sCr ratio in patients with renal impairment. These findings may have important implications for the clinical management of patients with systemic inflammation and concurrent renal impairment. However, the present findings may need to be validated in larger randomized prospective trials.

## 5. Conclusions

This study shows that the NGAL/sCr ratio is significantly associated with hsCRP and NLR levels. The NGAL/sCr ratio exhibits better diagnostic accuracy than NGAL in identifying hsCRP > 4.0 mg/dL in renal dysfunction. Measurement of the NGAL/sCr ratio may provide an additional benefit for monitoring patients with impaired renal function, particularly under inflammatory conditions.

## Figures and Tables

**Table 1:** Baseline characteristics of subject population included in this study.

	Patient populations (*n* = 162)	Healthy control (*n* = 45)	*P* value
Anthropometric parameters			
Age (years)	63 (32–81)	62 (31–79)	0.704
Gender (male, %)	86 (53.1)	24 (53.3)	0.989
BMI (kg/m^2^)	22.5 ± 3.5	23.3 ± 2.6	0.361
Systolic BP (mmHg)	126.4 ± 27.1	129.8 ± 16.7	0.485
Lipocalin levels			
NGAL (ng/mL)	186.5 (91.5–429.2)	60.0 (51.0–76.0)	<0.001
NGAL/sCr ratio	147.2 (81.5–261.2)	58.0 (51.0–76.0)	<0.001
Percent difference (95% CI)	9.1 (0.0–78.1)	0.0 (0.0–17.5)	<0.001
NGAL > 150 ng/mL (*n*, %)	92 (56.8)	0 (0.0)	<0.001
Inflammation indices			
hsCRP (mg/dL)	4.00 (1.93–11.23)	0.08 (0.04–0.16)	<0.001
hsCRP > 0.3 mg/dL (*n*, %)	162 (100.0)	0 (0.0)	<0.001
cESR (mm/h)	25.0 (12.4–45.2)	4.3 (1.9–6.4)	<0.001
Neutrophil (×10^9^/L)	6.57 (4.36–10.43)	4.09 (2.95–4.96)	<0.001
NLR	4.5 (3.0–12.1)	1.9 (1.2–3.1)	<0.001
Kidney function			
eGFR (mL/min/1.73 m^2^)	67.8 (45.4–85.7)	86.2 (77.2–95.3)	<0.001
sCr (mg/dL)	1.10 (0.81–1.50)	0.89 (0.80–0.97)	<0.001
sCr ≥ 1.2 mg/dL (*n*, %)	69 (42.6)	0 (0.0)	<0.001
BUN (mg/dL)	18.8 (12.1–28.8)	12.8 (10.7–15.5)	<0.001
Cardiac marker			
CK-MB (ng/mL)	2.8 (1.2–6.7)	2.7 (1.2–3.7)	0.392
Troponin-I (ng/mL)	0.1 (0.1–0.3)	0.1 (0.1–0.3)	0.781

Data are expressed as mean ± SD, median (interquartile range), or frequency (%).

BMI, body mass index; BP, blood pressure; NGAL, neutrophil gelatinase-associated lipocalin; NGAL/sCr ratio, NGAL-to-serum creatinine ratio; hsCRP, high sensitivity C-reactive protein; cESR, corrected erythrocyte sedimentation rate; NLR, neutrophil-to-lymphocyte ratio; eGFR, estimated glomerular filtration rate; sCr, serum creatinine; CK-MB, creatine kinase-MB.

**Table 2:** Plasma NGAL levels and NGAL/sCr ratios in relation to kidney function in patients with inflammation.

	Patients with inflammation		*P* value
	Without renal impairment (sCr < 1.2 mg/dL, *n* = 93)	With renal impairment (sCr ≥ 1.2 mg/dL, *n* = 69)	
Lipocalin levels			
NGAL (ng/mL)	125.0 (76.0–208.0)	417.0 (182.5–973.5)	<0.001
NGAL/sCr ratio	121.0 (71.7–204.9)	229.9 (100.2–320.3)	<0.001
Percent difference (95% CI)	0.0 (0.0–14.1)	36.7 (18.4–83.7)	<0.001
NGAL > 150 ng/mL (*n*, %)	37 (39.8)	55 (79.7)	<0.001
Inflammation indices			
hsCRP (mg/dL)	3.24 (0.98–8.41)	6.17 (3.21–14.76)	<0.001
cESR (mm/h)	20.9 (9.7–36.5)	32.8 (15.2–54.2)	0.004
Neutrophil (×10^9^/L)	6.17 (4.23–10.06)	7.05 (4.92–11.37)	0.095
NLR	3.8 (2.9–10.2)	5.7 (3.1–12.7)	0.037
Kidney function			
eGFR (mL/min/1.73 m^2^)	82.4 (72.4–101.4)	42.3 (23.3–53.7)	<0.001
sCr (mg/dL)	0.83 (0.74–1.03)	1.58 (1.31–2.55)	<0.001
BUN (mg/dL)	13.7 (10.9–19.4)	30.8 (20.5–49.7)	<0.001

Data are expressed as median (interquartile range) or frequency (%).

NGAL, neutrophil gelatinase-associated lipocalin; NGAL/sCr ratio, NGAL-to-serum creatinine ratio; hsCRP, high sensitivity C-reactive protein; cESR, corrected erythrocyte sedimentation rate; NLR, neutrophil-to-lymphocyte ratio; eGFR, estimated glomerular filtration rate; sCr, serum creatinine; BUN, blood urea nitrogen.

**Table 3:** Regression analysis of NGAL and NGAL/sCr ratios versus inflammatory parameters in patients with renal dysfunction.

Variables	NGAL/sCr ratio		NGAL	
	Univariate	Multivariate^*∗*^	Univariate	Multivariate^*∗*^
hsCRP (mg/dL)	0.591 (<0.001)	0.526 (<0.001)	0.487 (<0.001)	0.453 (<0.001)
cESR (mm/h)	0.454 (<0.001)	0.362 (<0.001)	0.267 (0.029)	0.230 (0.227)
Neutrophil (×10^9^/L)	0.392 (<0.001)	0.241 (0.132)	0.253 (0.038)	0.218 (0.254)
NLR	0.452 (<0.001)	0.428 (<0.001)	0.354 (<0.001)	0.327 (<0.001)

Correlations between inflammatory parameters and the levels of the NGAL/sCr ratio and NGAL are expressed as standard *β* (*P* value). ^*∗*^Multivariate: adjusted for age, BMI, systolic BP, hemoglobin, and troponin-I. NGAL, neutrophil gelatinase-associated lipocalin; NGAL/sCr ratio, NGAL-to-serum creatinine ratio; hsCRP, high sensitivity C-reactive protein; cESR, corrected erythrocyte sedimentation rate; NLR, neutrophil-to-lymphocyte ratio.

**Table 4:** Diagnostic ability of the NGAL/sCr ratio to identify NLR > 4.5 and cESR > 25 mm/h in patients with renal dysfunction, compared with that of NGAL and hsCRP.

	ROC curve analysis		
	AUC	95% confidence interval	*P* values
*NGAL versus NGAL/sCr ratio *			
NLR > 4.5			
NGAL/sCr ratio	0.674	0.545–0.803	0.016
NGAL	0.623	0.482–0.763	0.048
cESR > 25 mm/h			
NGAL/sCr ratio	0.686	0.556–0.817	0.013
NGAL	0.635	0.487–0.785	0.041

*hsCRP versus NGAL/sCr ratio *			
NLR > 4.5			
NGAL/sCr ratio	0.729	0.594–0.865	0.002
hsCRP	0.665	0.517–0.815	0.027
cESR > 25 mm/h			
NGAL/sCr ratio	0.686	0.560–0.812	0.010
hsCRP	0.674	0.545–0.803	0.016

ROC, receiver operating characteristics; AUC, area under the curve; NGAL, neutrophil gelatinase-associated lipocalin; NGAL/sCr ratio, NGAL-to-serum creatinine ratio; NLR, neutrophil-to-lymphocyte ratio; cESR, corrected erythrocyte sedimentation rate; hsCRP, high sensitivity C-reactive protein.
